# Impact of the Number of Needle Tip Bevels on the Exerted Forces and Energy in Insulin Pen Injections

**DOI:** 10.3390/s23198043

**Published:** 2023-09-23

**Authors:** Alfonso Maria Ponsiglione, Carlo Ricciardi, Enzo Bonora, Francesco Amato, Maria Romano

**Affiliations:** 1 Department of Electrical Engineering and Information Technology, University of Naples Federico II, 80125 Naples, Italy; alfonsomaria.ponsiglione@unina.it (A.M.P.); framato@unina.it (F.A.); mariarom@unina.it (M.R.); 2 Division of Endocrinology, Diabetes and Metabolism, University and Hospital Trust of Verona, 37129 Verona, Italy; enzo.bonora@univr.it

**Keywords:** diabetes mellitus, insulin pen, injection–extraction cycle, insulin injection, needles

## Abstract

Patients affected with type 1 diabetes and a non-negligible number of patients with type 2 diabetes are insulin dependent. Both the injection technique and the choice of the most suitable needle are fundamental for allowing them to have a good injection experience. The needles may differ in several parameters, from the length and diameter, up to the forces required to perform the injection and to some geometrical parameters of the needle tip (e.g., number of facets or bevels). The aim of the research is to investigate whether an increased number of bevels could decrease forces and energy involved in the insertion–extraction cycle, thus potentially allowing patients to experience lower pain. Two needle variants, namely, 31 G × 5 mm and 32 G × 4 mm, are considered, and experimental tests are carried out to compare 3-bevels with 5-bevels needles for both the variants. The analysis of the forces and energy for both variants show that the needles with 5 bevels require a statistically significant lower drag or sliding force (*p*-value = 0.040 for the 31 G × 5 mm needle and *p*-value < 0.001 for 32 G × 4 mm), extraction force (*p*-value < 0.001 for both variants), and energy (*p*-value < 0.001 for both variants) during the insertion–extraction cycle. As a result, 3-bevels needles do not have the same functionality of 5-bevels needles, show lower capacity of drag and extraction, and can potentially be related to more painful injection experience for patients.

## 1. Introduction

According to the World Health Organization [1], diabetes, also referred to as diabetes mellitus, is a chronic disease characterized either by a deficient production of insulin or by an ineffective use of insulin. As a consequence of uncontrolled diabetes, the increase in blood glucose level, also termed as hyperglycemia, can severely damage systems and organs, e.g., nerves and blood vessels, thus increasing the risk of developing cardiovascular diseases and other microvascular complications [2,3,4,5]. In 2021, it was estimated that almost 540 million adults (20–79 years) were affected with diabetes, with about 2 million deaths per year; moreover, more than one million children and adolescents are affected with type 1 diabetes [6,7].

In 1936, the scientist Harold Himsworth [6] described for the first time the difference between the two main forms of diabetes: type 1 and type 2. On the one hand, in type 1 diabetes (or insulin-dependent diabetes), the pancreas is not able to produce enough insulin to lower the level of glucose in the blood due to the destruction of insulin-producing pancreatic β cells [7,8]. On the other hand, type 2 diabetes is characterized by insulin resistance, i.e., the inability of insulin-sensitive tissues to respond appropriately to insulin, and may occur due to obesity, hypertension, and dyslipidemia [9,10]. While type 2 diabetes usually does not require insulin-based treatments [11,12], for patients affected with type 1 diabetes, as no cure currently exists, the only possible therapeutic approach consists of a lifelong exogenous insulin replacement therapy (with multiple daily injections [13,14].

The introduction of disposable insulin-specific syringes brought several improvements in terms of higher usability [15,16]. Then, the introduction of “pens” (a disposable or reusable instrument) yielded other advantages, such as better dosage control, reduced pain and plunger force to facilitate the administration of larger doses of insulin, and better acceptance [15,16].

Using insulin pens is fundamental to effectively administer insulin to the body [17]. In particular, the quality of insulin needles translates substantially into technological innovation, as this can determine both a greater effectiveness of the treatment and a better quality of life for patients [18]. Indeed, some pen needles have been formulated to have special edges that may yield a smoother and gentler injection.

With the purpose of facilitating the insertion into the skin and, at the same time, maximizing the insulin delivery and reducing the tissue damage and the related pain perceived by the patient, insulin needles’ manufacturers face the challenge of reducing the needle diameter and length, while preserving the inner lumen dimensions in order to ensure a proper insulin flow and delivery [19], in accordance with national and international diabetes guidelines [20,21]. Therefore, insulin needles are mainly characterized and sold according to their length and diameter (often measured in gauge, G) as they can, directly or indirectly, determine some relevant technical parameters, such as the force required to perform the injection [22] or the flow of the drug through the needle [23]; moreover, they can affect some patient-related factors, such as preference [24], adherence [25], pain perception [26], and glycemic control [18].

Concerning the length and gauge, the standards of care of the Italian Society of Diabetology define the 32 G × 4 mm needle as the best choice for a patient (gold standard), as it guarantees optimal insulin absorption and causes less pain [21]. As for the injection angle, it is generally recommended to place the needle with a 90° angle with respect to the skin; even though, in specific cases, the injection angle can be lower, up to 45° [27]. In addition, from the scientific debate, it emerges that, at fixed length and gauge of the needle, the tip geometry (the number of facets/bevels and the shape of the tip) is among the most relevant parameters characterizing the needles for insulin administration. Indeed, the sharpness of the needle tip, which is dependent on the number of tip bevels, could influence both the injection forces and the energy transferred to the tissue, thus determining the magnitude of pain perceived by the patient, although this issue has not been fully explored in the literature so far [28,29].

On these premises, the aim of this work is to exactly investigate the latter issue and that is to rigorously show that the tip geometry plays a fundamental role in determining the amount of force and energy involved in the injection process, thus determining the intensity of the pain suffered by the patient.

In this context, we shall show that the use of 5-bevels needles can decrease both the involved forces and the energy transferred during the insertion–extraction cycle with respect to 3-bevels needles. For this purpose, following a theoretical discussion, an experimental study has been conducted, as described in the following sections. The experiments confirm the premise of the theory, since 5-bevels needles clearly exhibit a better performance in terms of the forces generated and the energy transferred during the whole process.

## 2. Materials and Methods

### 2.1. The Insertion–Extraction Cycle

Different from the literature, which usually focuses on the single forces involved during the injection process, here the full needle insertion–extraction cycle has been examined as a quantitative descriptor of the whole injection experience.

In the injection–extraction cycle, the following three main forces have to be considered:

- Penetration Force (PF): the maximum force needed by the tip of a lubricated needle to penetrate the skin (the greater the force, the greater the perceived pain).
- Drag force (DF): the frictional force that the needle encounters during the “sliding” of the needle into the skin to reach the injection site (the greater the force, the greater the tissue trauma and perceived pain).
- Extraction force (EF): the frictional force that the needle encounters during the “sliding” phase of the needle during the leakage from the tissue (the greater the force, the greater the trauma to the tissues and the perceived pain).

**Figure 1:**
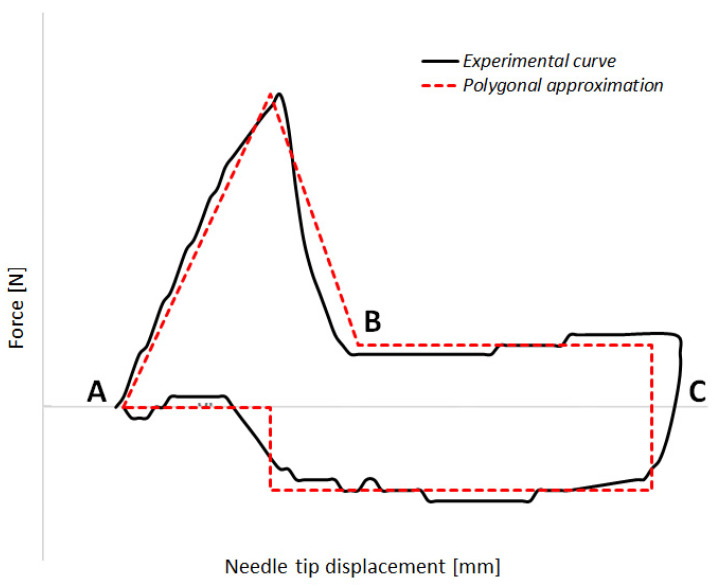
Needle insertion–extraction cycle: forces are plotted against needle displacement (black line represents the experimental data; red dashed line represents a polygonal approximation considering a polygonal shape whose vertices are the maximum force at 25% of needle length, the drag force between 40% and 90% of length, and the extraction force between 90% and 25% of length). The first section of the graph (AB) depicts the penetration phase, the second one (BC) displays the insertion phase, and finally, the third one (CA) represents the extraction phase.

Figure 1 shows an example of the needle insertion–extraction cycle, together with a schematic polygonal decomposition of the experimental curve to better display the different phases of the cycle, where the *x*-axis represents the displacement of the needle (measured in mm), and the *y*-axis represents the force (expressed in N). In Figure 1, the first section of the graph (AB) depicts the penetration phase, the second one (BC) displays the insertion phase, and finally, the third one (CA) represents the extraction phase. The forces during the insertion (BC) and extraction (CA) phases are essentially the same, but are in the opposite direction.

### 2.2. Energy as Expression of Perceived Pain

Starting from the previous results, the energy released by the needle to the substrate during the test is estimated. Assuming that the forces, especially the DF and the EF, are correlated with the needle–tissue friction, which could then cause pain to the patient, we can conclude that the energy of the insertion–extraction cycle is a proxy for the perceived pain, as it represents the dissipated energy that is transferred to the subcutaneous tissues (thus causing pain, hematomas, etc.). In this sense, the energy is chosen here as a further meaningful indirect indicator of the perceived pain during the injection procedure.

It is well known that the Energy can be expressed as the following integral of the infinitesimal work *F*(*x*)*dx*:

(1) $$\mathrm{Energy}=\int_{\mathrm{insertion}-\mathrm{extraction}\;\mathrm{cycle}\;}^{}F\left(x\right)dx$$

Therefore, Equation (1), representing the area within the red dashed curve in Figure 1, refers to the Energy (in mJ) transmitted from the needle to the substrate.

### 2.3. The Curve Profile

In this section, we will show why a needle with a greater number of bevels is useful in reducing pain in patients.

The lower the angle between the first trait of the needle and the needle itself, the lower the PF, and that implies a dependence of the pain on the needle tip. DF and EF are increasing with the friction forces; again, the lower the angles between the various traits of the needle, the lower the friction forces. Therefore, the perceived pain will be less when the tip of the needle does not have angularities that form very sharp angles, as it can be assumed that a higher potential tissue damage is related to a sharper angle.

A needle profile with a greater number of bevels allows a decrease in the slope of each needle section compared to the contiguous one, thus decreasing the DF and the EF. According to these considerations, a continuous “curve” profile should be the best option, because it minimizes (ideally reduces to zero) at each point in the angle between the tangent to the profile and the needle axis. 

**Figure 2:**
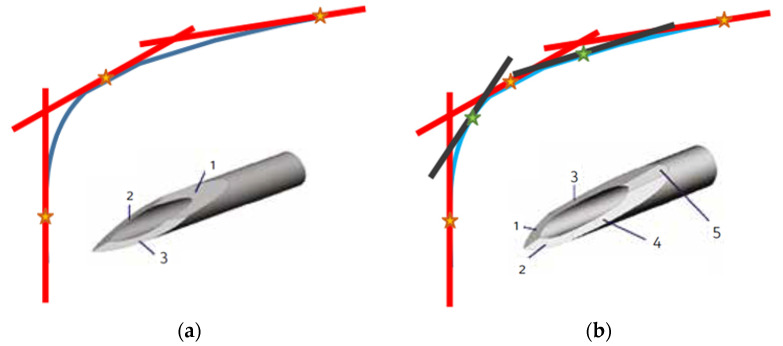
Curvature approximation with (**a**) three and (**b**) five segments.

In order to approximate the ideal curve profile, which can be interpreted as the plot of a given function of the *x* variable, we can resort to a truncated Taylor series, as it allows summing a finite number of terms. With the aim of approximating a curve profile by using a limited number of linear segments, it is worth noting that, as the number of terms (segments) increases, a more accurate approximation of the curve is obtained, as illustrated in Figure 2.

Indeed, Figure 2 clearly illustrates how the increase in the number of segments (i.e., the number of bevels in the case of needles) allows to obtain a smoother profile (i.e., more obtuse angles between the segments exploited to approximate the curve).

### 2.4. Experimental Study

An experimental study has been conducted with the aim of estimating and comparing penetration, drag, and extraction forces of pen needles with different number of bevels. Furthermore, based on the performed tests, the energy involved in the insertion–extraction cycle has been computed.

The test procedure, to determine penetration, drag, and extraction forces of pen needles, is based mainly on indications from Annex D of the ISO 7864 norm [30].

Each test has been based on the following protocol:

- The substrate strip, made of a 1 mm thick natural latex rubber and with a 45 Shore A hardness, is moved 10 mm far, tensioned and clamped.
- The needle to test is screwed on the threaded support and fixed to the tensile test machine (MTS Alliance RT/10, RTM Code 00782), then positioned at a fixed distance from the substrate.
- The machine starts in displacement control far enough to impact substrate at a 100 mm/min constant velocity, after it decelerates to stop when at least 80% of length is in. It starts back, to reach 100 mm/min and to exit completely from the substrate.
- The needle displacement is continuously recorded together with force, measured with a 100 N load cell.

Two needle variants, namely, 31 G × 5 mm and 32 G × 4 mm, are considered, and experimental tests are carried out to compare 3-bevels with 5-bevels needles for both the variants. For each test, 50 measurements were conducted on pen needles randomly drawn by three different lots. The measurements have been fully randomized to mitigate any potential test bias.

Concerning the force measurements, it is worth mentioning that, in the case of the 31 G × 5 mm needle variant (either with 3- or 5-bevels), since most needles showed a stable DF and EF along a 2 mm path, the mean value to calculate such forces was taken between 40% and 80% of the needle length (that is from approx. 2 to 4 mm of the displacement, starting from the contact with the substrate of 5 mm long items). In the case of 32 G × 4 mm needle variant (either with 3- or 5-bevels), as most needles showed a stable DF and EF along a 1 mm path, the mean value to calculate such forces was taken between 55% and 80% of the needle length (that is from approx. 2.2 to 3.2 mm of the displacement, starting from the contact with the substrate, comprising 4 mm long items).

Regarding the energy estimation, it is worth mentioning that, in the case of the 31 G × 5 mm needle variant (either with 3- or 5-bevels), the energy transmitted from the needle to the substrate (i.e., the area inside the experimental curve) and is approximated by the area of a polygon whose vertices are the maximum force at 25% of the needle length, DF between 40% and 90% of the length, and EF between 90% and 25% of the length. In the case of the 32 G × 4 mm needle variant (either with 3- or 5-bevels), the polygon has its vertices corresponding to the maximum force at 35% of needle length, DF between 35% and 90% of the length, and EF between 90% and 30% of the length. The polygonal decomposition of the measured force–displacement data was employed as it allows better representation of the different phases of the injection process, with the different segments of the polygonal curve are clearly discernible (sections AB, BC, and CA). Moreover, it provides easier and reliable estimates of the energy, without the need for more complex numerical integration methods and without introducing uncertainties higher than the measurement uncertainty itself.

### 2.5. Statistical Analysis and Results Comparison

The comparison between the forces and the energies during the insertion–extraction cycle has been conducted by applying the paired Student’s *t*-tests for those samples with normal distribution, and a Wilcoxon signed-rank test as a nonparametric test equivalent to the dependent *t*-test for non-normally distributed data. Normality check has been carried out by means of the Shapiro–Wilk test. For statistical tests, the confidence interval was set to 95% (significance level α = 0.05). All the tests have been performed by using the IBM SPSS Statistics for Data Analysis v.27.

In addition to the descriptive statistics and to the hypothesis tests, two additional parameters to describe the relative changes in both force and energy metrics have been considered:

(2) $$\Delta Force\%=\left(\frac{{\bar{F}}_{3}-{\bar{F}}_{5}}{{\bar{F}}_{5}}\right)\times 100$$

(3) $$\Delta Energy\%=\left(\frac{{\bar{E}}_{3}-{\bar{E}}_{5}}{{\bar{E}}_{5}}\right)\times 100$$

where

- ${\bar{F}}_{3}$ represents the average value of the exerted force (either PF, DF, or EF) in the case of 3-bevels needle;
- ${\bar{F}}_{5}$ represents the average value of the exerted force (either PF, DF, or EF) in the case of 5-bevels needle;
- ${\bar{E}}_{3}$ represents the average value of the energy of the insertion–extraction cycle in the case of 3-bevels needle;
- ${\bar{E}}_{5}$ represents the average value of the energy of the insertion–extraction cycle in the case of 5-bevels needle.

The parameters Δ*Force*% and Δ*Energy*% represent the average percentage difference in forces (either PF, DF, or EF) and energy that characterize the insertion–extraction cycle in the case of a 3-bevels insulin pen needle with respect to the case of a 5-bevels insulin pen needle, respectively.

## 3. Results

Experimental tests were conducted on two variants (31 G × 5 mm and 32 G × 4 mm) for both 3-bevels insulin pen needles from Pikdare S.p.A. (Como, Italy) and 5-bevels insulin pen needles from Becton Dickinson. The analysis of forces and energy is presented in the following subsections, which illustrate the results obtained from the test described in the experimental section and drive the subsequent statistical analysis, carried out to compare the characteristic parameters of the injection–extraction cycle between 3-bevels and 5-bevels pen needles.

### 3.1. Insertion–Extraction Cycle Results

**Figure 3:**
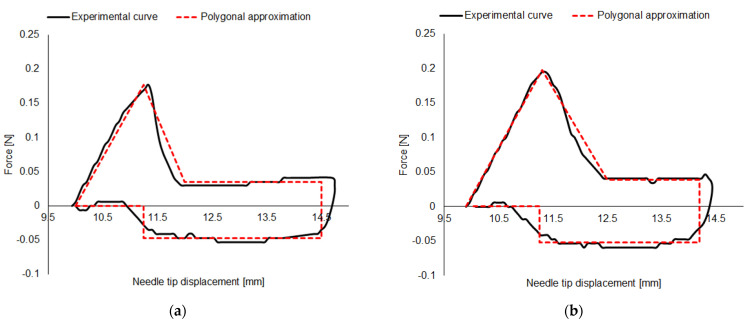

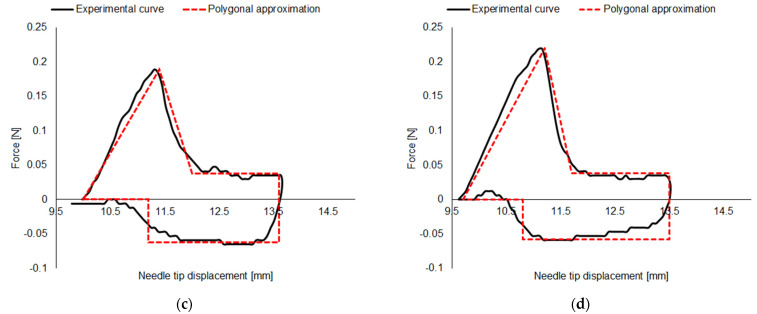
Insertion–extraction cycle on (**a**) 5−bevels 31 G × 5 mm needle; (**b**) 3−bevels 31 G × 5 mm needle; (**c**) 5−bevels 32 G × 4 mm needle; and (**d**) 3−bevels 32 G × 4 mm needle.

Figure 3 displays a typical insertion–extraction cycle curve, together with its polygonal fit, obtained for both variants and for both numbers of bevels tested.

From a preliminary visual inspection, while no considerable differences in the maximum and minimum values of the forces can be detected, due to the reduced dimensions, the 32 G × 4 mm needle variant shows a shorter displacement range.

Details and analysis carried out on the forces and energies extracted by each measured insertion–extraction cycle are presented in the following sections.

### 3.2. Force Analysis

**Figure 4:**
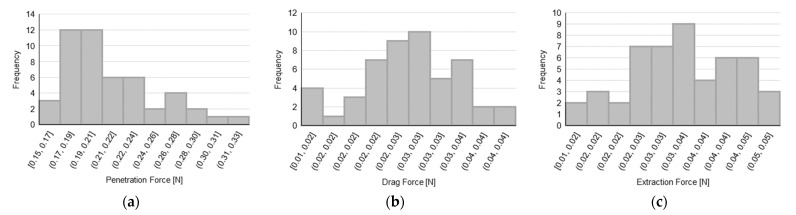

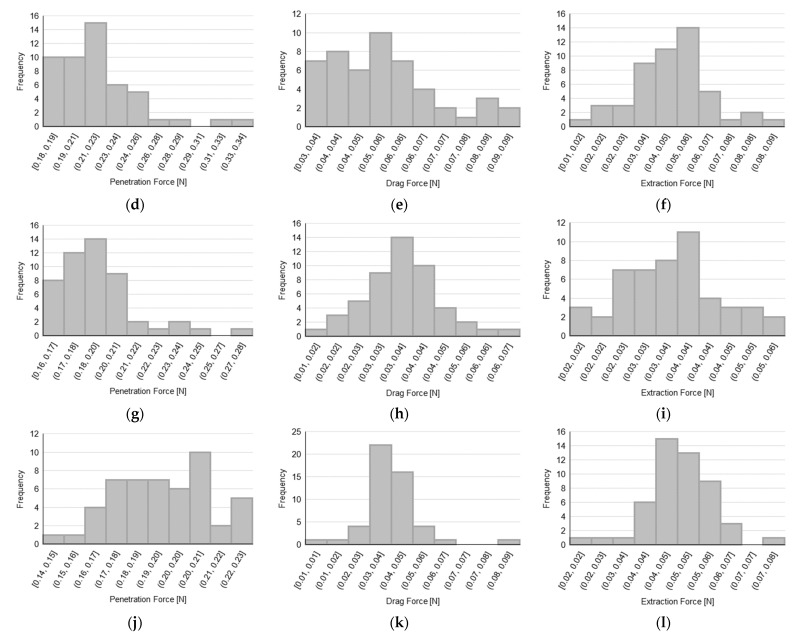
Force data distributions for (**a**–**c**) PF, DF, and EF for 31 G × 5 mm needle with 5-bevels; (**d**–**f**) PF, DF, and EF for 31 G × 5 mm needle with 3-bevels; (**g**–**i**) PF, DF, and EF for 32 G × 4 mm needle with 5-bevels; and (**j**–**l**) PF, DF, and EF for 32 G × 4 mm needle with 3-bevels.

Data distributions for PF, DF, and EF are presented in Figure 4 for each tested sample with the aim of assessing the normality of the distributions, before performing the hypothesis tests to compare 3-bevels with 5-bevels needles.

In order to establish the normality of the data based on the observed distributions, a Shapiro–Wilk test has been carried out and the corresponding results are reported in Table 1, which describes, for each needle type and for each force parameter, both the value of the W-statistic of the test with the number of tests performed, and the significance of the test in terms of *p*-value (a *p*-value lower than 0.05 indicates a non-normal distribution). We recall that the W-statistic represents the difference between the normal distribution model and the observations (with relatively smaller values of the W-statistic indicating a worse fit of the sample data compared to a normal distribution).

Moreover, Table 2 shows the results in terms of the forces exerted during the insertion–extraction cycle on a collection of 50 test measurements carried out for each needle variant and according to the number of bevels.

The analysis of the forces for both variants show that DF and EF are significantly lower for needles with 5-bevels with respect to those ones with 3-bevels (*p*-value < 0.001 for the DF in the 31 G × 5 mm variant, *p*-value = 0.040 for the DF in 32 G × 4 mm variant, and *p*-value < 0.001 for the EF in both variants). As far as the PF is concerned, it does not show a statistically significant difference between the needles with 5- and 3-bevels. This results from the fundamental difference between the type of forces involved in the insertion–extraction cycle. Indeed, while the PF is a pointwise value representing the peak force at the insertion phase (calculated as the maximum value of the force), both DF and EF are average values (calculated as a mean along an approximate path of 2 mm) taking into account the longer duration and higher friction exerted on the subcutaneous tissue by the needle sliding in both the insertion and extraction directions. Thus, the values of DF and EF can better explain the difference in the needle tip characteristics (i.e., the number of bevels) during the injection process. Furthermore, it is also worth noting that there is always a positive average percentage difference between the 3-bevels needle and the 5-bevels one, thus confirming that the use of a 3-bevels needle produces an increase in all the exerted forces in the insertion–extraction cycle. This is observable for both needle variants, with a maximum increase of almost 90% in the DF for the 31 G × 5 mm needle.

**Figure 5:**
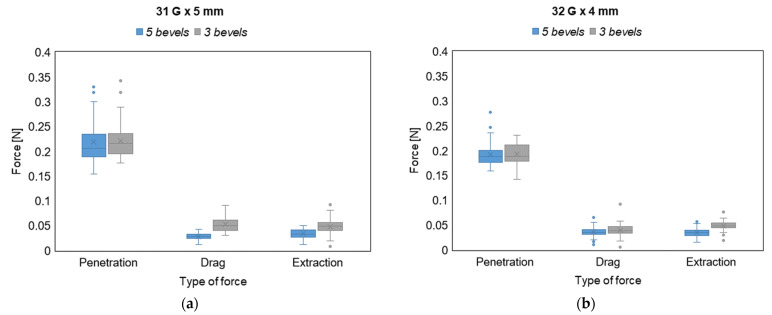
Force values boxplots for (**a**) 31 G × 5 mm needle and (**b**) 32 G × 4 mm needle. Extreme outliers (i.e., those exceeding the first, or the third, quartile of the distribution by three times the interquartile range) are marked with asterisks, mean values are marked with an × within the boxplot, and median values are reported with a straight line within the boxplot.

Figure 5 provides a visual representation of the difference between the exerted forces during the whole injection process, with 3- and 5-bevels needles, in both sample variants.

From the boxplots displayed in Figure 5, it can be observed how the PF, despite not showing significant differences between 3- and 5-bevels, exhibits the largest absolute values and the largest intra-sample variability compared to the other types of forces. In addition, it can be also noted how the ranges of variability of the forces do not significantly change between the two variants tested.

### 3.3. Energy Analysis

As the perceived pain is dependent on the forces, it is possible to consider that the energy of the insertion–extraction cycle is representative of the overall perceived pain during the whole injection process. Based on the forces measurements previously presented and relying on Equation (1), the insertion–extraction cycle allows the estimation of the energy involved in the whole injection process.

**Figure 6:**
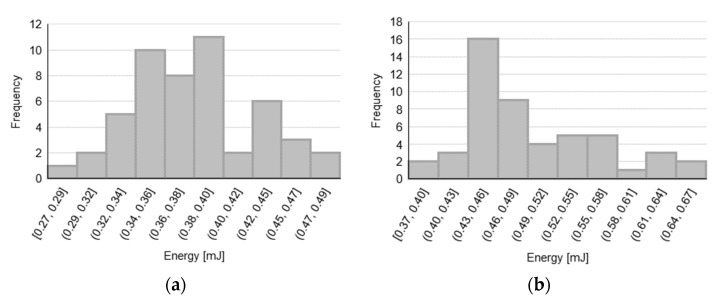

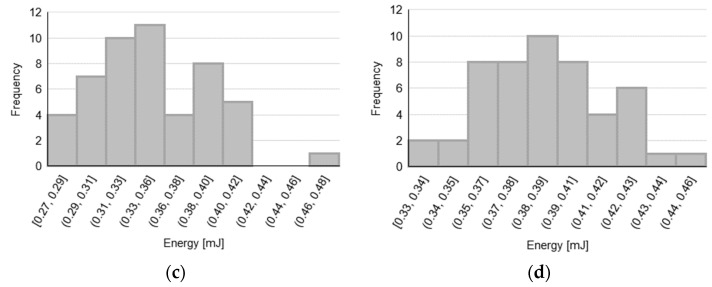
Energy data distributions for (**a**) 31 G × 5 mm needle with 5-bevels; (**b**) 31 G × 5 mm needle with 3-bevels; (**c**) 32 G × 4 mm needle with 5-bevels; and (**d**) 32 G × 4 mm needle with 3-bevels.

First, the data distributions for energy estimates for each tested sample are observed (see Figure 6) in order to assess the data normality, before proceeding with the hypothesis tests to compare the 3-bevels with the 5-bevels needles.

Then, similar to the forces analysis, the normality of the observed distributions for the energy estimates has been determined by means of the Shapiro–Wilk test, whose results are reported in Table 3 (a *p*-value lower than 0.05 indicates a non-normal distribution).

Table 4 shows the results of the test conducted on both variants regarding the energies generated during the insertion–extraction cycle on the 50 measures taken for each needle variant.

The analysis of the energies for both variants show that the needles with 5-bevels require a statistically significant lower energy during the insertion–extraction cycle (*p*-value < 0.001). In particular, the analysis of the energy for both variants show a significant average percentage increase in the energy of the whole injection process when using the 3-bevels needle, with a 29% and 11% increase for the 31 G × 5 mm and the 32 G × 4 mm needle variants, respectively. Based on the analysis of the forces presented in the previous subsection, since the effects of the DF and the EF can reasonably explain the difference between the two types of needle tips, we can conclude that the energy increase is mainly due to the energy dissipated during the two sliding phases (i.e., the drag and extraction actions) of the cycle, that is, when the friction between the needle and the tissues occurs. As a results, the 3-bevels needle is confirmed as the most expensive choice also in energy terms.

**Figure 7:**
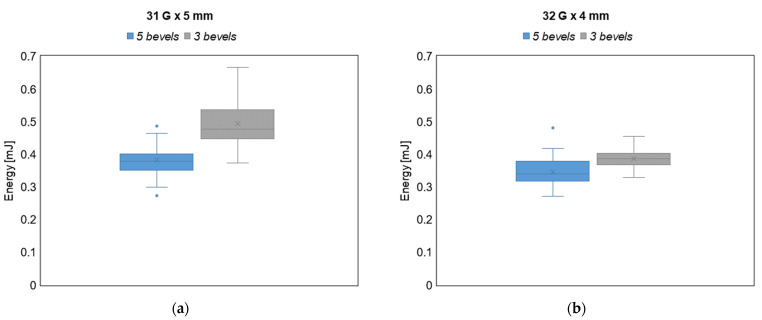
Energy values boxplots for (**a**) 31 G × 5 mm needle and (**b**) 32 G × 4 mm needle. Extreme outliers (i.e., those exceeding the first, or the third, quartile of the distribution by three times the interquartile range) are marked with asterisks, mean values are marked with an × within the boxplot, and median values are reported with a straight line within the boxplot.

Finally, in Figure 7, a visual representation of the difference between the energy involved in the insertion–extraction cycle is provided for both the 3- and the 5-bevels needles and for both sample variants.

By observing the boxplots in Figure 7, it is evident that, in the case of the 5-bevels needle, both the energy ranges and the intra-sample variability do not change across the two needle variants, thus showing greater stability in terms of energy dissipation with respect to the 3-bevels needle, which instead exhibits a significant variability across the needle variants. Furthermore, 5-bevels needle shows a statistically significant reduction in terms of energy compared to 3-bevels needles in both variants, thus confirming that the increased number of bevels is an energetic favorable choice.

Taken together with the numeric results showed in Table 4, the energy reduction brought by the 5-bevels needle tip, which is around 11% in the case of the 32 G × 4 mm variant, is even more marked for the 31 G × 5 mm variant (reaching a 29% reduction).

## 4. Discussion

Insulin therapy is the standard therapy for patients with type 1 diabetes, and it is the recommended course of treatment for some patients with type 2 diabetes. Since injections under the skin have been linked to pain, discomfort, and anxiety, pens for administering insulin, with shorter needles, have been chosen as an alternative to vials and syringes, because they can cause less pain and reduce skin harm. Alternatively to the insulin pen, patients can adopt insulin pumps, small electronic instruments that are connected to a cannula and a small plastic catheter, placed under the skin of the abdomen and replaced periodically, and administer insulin continuously [31].

It is known that the needle diameter and length directly influence the perceived pain. The tip shape, sharpness, insertion angle, glide, and the number of friction points (i.e., the number of bevels) are crucial factors in maximizing a needle’s ability to penetrate tissue and boosting patients’ acceptability. Due to their importance, the use of insulin pen needles has been largely studied in the literature [32], also focusing on further and less explored characteristics of both the needle and the insulin administration process. For example, Hirsch et al. [24] examined injection forces, self-reported patients pain, and preferences in people with diabetes. Other studies focused on the influence of needle lubrication [33] or injection volumes [34] on the perceived pain. The optimal location for the injection site has been also investigated and proved to be a relevant factor in the injection process, as different absorption rates are associated with different sites (e.g., abdomen, arms, and thighs) [35,36]. Later, Praestmark et al. [22] used 3-bevel asymmetrically modified needle tips that demonstrated better performance than traditional grind 3-bevels needles in terms of forces and pain; however, they did not focus on the energy characterizing the overall injection process.

As already highlighted in the introduction, the quality of insulin needles has a significant impact on both treatment efficacy and quality of life, thus potentially contributing to a decrease in health expenditures. However, there are still a few studies focused on the systematic analysis of the impact of specific needle tip characteristics, such as the number of bevels, on the injection process and on the perceived pain.

In this framework, this paper investigated and compared two variants of needles (31 G × 5 mm and 32 G × 4 mm), by means of an extensive statistical approach, considering several samples from different lots in order to estimate the forces and the energy during the injection–extraction cycle of needles with 3- and 5-bevels. Our data have shown that the DF and EF are significantly lower in 5-bevels needles (independent of the variant) as well as the overall energy associated with the cycle.

The most significant results are in line with the literature. In a survey conducted by Aronson et al. [37], it is stated that 5-bevels had a better performance than 3-bevels needles with respect to PF, DF, and perceived pain; however, while the work mainly focuses on needle length and diameter as well as on the injection context, no mention is made on the energy involved in the injection process. Moreover, a study by the same authors aimed at assessing the influence of a new extra-thin wall needle versus a usual one on the overall patient preference, ease of injection, perceived time to complete the full dose, thumb button force to deliver the injection, and dose delivery confidence in individuals with diabetes mellitus [38]. Although these considerations are not directly related to the study of the needle profile considered in this paper, they do show that the quality of needles with a greater number of bevels is superior under all aspects.

As mentioned in the introduction, Praestmark et al. demonstrated that the standard 3-bevels 32 G needle caused a larger peak PF than the asymmetrical 3-bevels tapered 34 G and the 5-bevels 32 G needles [22]. Similarly, preclinical force testing in a laboratory measured 23% less mean PF for 5-bevel needles when compared to similar-sized 3-bevel needles (from 32 G × 4 mm to 31 G × 8 mm) [24]. After additional studies, the authors hypothesized that the 5-bevel needle tip may support better acceptance of self-injection therapy.

The present work offers an additional novel contribution to the previous literature by introducing and investigating a less explored but relevant parameter from an engineering perspective, i.e., the involved force and energy, chosen as indirect indicators of the perceived pain. This paper describes a route for the comparison of 3-bevels and 5-bevels insulin pen needles by means of a theoretical–mathematical approach together with an experimental study to test the formulated hypotheses, based on the analysis of the whole insertion–extraction cycle, to describe the whole injection experience. The obtained results strongly supported and confirmed the hypotheses made regarding the advantages of increasing the number of bevels in the insulin needle tips and provided new insights in the comprehension of the role of the different forces involved in the injection process and on the leading role of the energy as a potential expression of the perceived pain. In particular, according to the data shown in this paper, we can state that 3-bevels needles do not have the same performance of 5-bevels needles and show lower capacity of drag and extraction. The same can be stated for the energy parameter (taken as a proxy of the perceived pain) as 5-bevels needles can decrease both forces and energy during the insertion–extraction cycle with respect to 3-bevels needles, thus potentially allowing patients to experience reduced pain.

Since the experimental study has been conducted on artificial skin equivalents, further improvements and advancements will be required in future works to provide a clinical validation of the experimental results obtained here.

## 5. Conclusions

There are several crucial characteristics of insulin pen needles with an immediate impact on the injection process, patient adherence, and quality of life. To improve the patient experience and lessen the discomfort associated with the injection, forces involved in the injection process are significant factors as well as other geometric parameters like the gauge and the length of the needle. In this work, we focus on the tip profile, to show that it can have a strong impact on the magnitude of the pain perceived by the patient; in particular, we showed that pen needles with 5-bevels result in a lower pain experience, by demonstrating a decrease in both the involved forces and the transferred energy during the insertion–extraction cycle of the two needle variants (31 G × 5 mm and 32 G × 4 mm) by means of a theoretical development and an experimental study followed by an in-depth statistical analysis. The results demonstrate that 3-bevels needles do not have the same performance as that of 5-bevels needles and show lower capacity of drag and extraction and cause higher pain to patients. Features like the number of bevels in pen needles can therefore represent a significant factor in determining the effectiveness and patient acceptability towards insulin injection treatments and can guide the development of novel needles that are more responsive to patient demands.

## Figures and Tables

**Table 1:** Results of the Shapiro–Wilk normality tests on PF, DF, and EF measurements.

Variant	Number of Bevels	Forces	Statistic	Number of Tests	*p*-Value
31 G × 5 mm	5-bevels	PF	0.914	50	**0.001**
		DF	0.985	50	0.766
		EF	0.975	50	0.365
	3-bevels	PF	0.890	50	**0.000**
		DF	0.938	50	**0.012**
		EF	0.963	50	0.114
32 G × 4 mm	5-bevels	PF	0.877	50	**0.000**
		DF	0.974	50	0.343
		EF	0.988	50	0.884
	3-bevels	PF	0.972	50	0.285
		DF	0.842	50	**0.000**
		EF	0.956	50	0.060

**Table 2:** Comparisons between forces of 31 G × 5 mm and 32 G × 4 mm needles according to the number of bevels.

Variant	Number of Tests	Forces	Metrics	5-Bevels	3-Bevels	ΔForce%	*p*-Value
31 G × 5 mm	50	PF	Avg.	0.218	0.221	1.38	0.647 **^1^**
			Std. Dev.	0.040	0.034		
			Max	0.331	0.343		
			Min	0.154	0.177		
		DF	Avg.	0.028	0.053	89.3	**0.000 ^1^**
			Std. Dev.	0.007	0.015		
			Max	0.043	0.091		
			Min	0.012	0.031		
		EF	Avg.	0.034	0.048	41.2	**0.000 ^2^**
			Std. Dev.	0.010	0.015		
			Max	0.051	0.092		
			Min	0.012	0.008		
32 G × 4 mm	50	PF	Avg.	0.192	0.193	0.52	0.537 **^1^**
			Std. Dev.	0.022	0.021		
			Max	0.278	0.231		
			Min	0.160	0.142		
		DF	Avg.	0.036	0.040	11.1	**0.040 ^1^**
			Std. Dev.	0.010	0.012		
			Max	0.066	0.092		
			Min	0.011	0.006		
		EF	Avg.	0.036	0.049	36.1	**0.000 ^2^**
			Std. Dev.	0.009	0.009		
			Max	0.057	0.077		
			Min	0.016	0.019		

^1^ Wilcoxon signed-rank test; ^2^ Paired Student’s *t*-test.

**Table 3:** Results of the Shapiro–Wilk normality tests on energy estimates.

Variant	Number of Bevels	Statistic	Number of Tests	*p*-Value
31 G × 5 mm	5-bevels	0.983	50	0.664
	3-bevels	0.938	50	**0.011**
32 G × 4 mm	5-bevels	0.972	50	0.272
	3-bevels	0.990	50	0.946

**Table 4:** Comparison between energies of the 31 G × 5 mm and 32 G × 4 mm needles according to the number of bevels.

Variant	Metrics	5-Bevels	3-Bevels	ΔEnergy%	*p*-Value
31 G × 5 mm	Avg.	0.383	0.494	29.0	**0.000 ^1^**
	Std. Dev.	0.047	0.069		
	Max	0.488	0.667		
	Min	0.273	0.374		
32 G × 4 mm	Avg.	0.347	0.387	11.5	**0.000 ^2^**
	Std. Dev.	0.043	0.027		
	Max	0.482	0.456		
	Min	0.272	0.329		

^1^ Wilcoxon signed-rank test; ^2^ Paired Student’s *t*-test.

## Data Availability

Not applicable.
